# Characterization of Cryopreserved Canine Amniotic Membrane

**DOI:** 10.3390/membranes11110824

**Published:** 2021-10-27

**Authors:** Nathawan Withavatpongtorn, Nalinee Tuntivanich

**Affiliations:** Faculty of Veterinary Science, Chulalongkorn University, 39 Henry Dunant Rd, Pathumwan, Bangkok 10330, Thailand; nathawan.withavat@gmail.com

**Keywords:** dogs, amnion, cryopreservation, ophthalmology, biomechanical phenomena

## Abstract

Amniotic membrane is an effective corneal reconstruction material in veterinary surgery. Cryopreserved amniotic membrane is widely used in practice. Properties of cryopreserved canine amniotic membranes are currently not well studied. This study aimed to compare three properties between canine amniotic membranes cryopreserved for 7 days and 30 days, including tensile strength, transparency, and cell viability. After their respective cryopreservation time, stress–strain curves of the cryopreserved membranes’ tensile strength were assessed using a universal testing machine. Both groups produced J-shaped stress–strain curves with statistically comparable parameters, including maximum stress, strain, and Young’s modulus. The percentage of cell viability was observed by trypan blue staining under a light microscope. Membrane transparency was tested with a spectrophotometer. Transparency tests showed high levels of light transmission and low haze, with no statistical difference between groups. Cell viability was statistically lower in the 30-day cryopreserved group. Tensile strength and transparency of cryopreserved CAM were not significantly impeded for up to 30 days. For CAM to be used as an alternative corneal transplant material in veterinary and regenerative medicine, further research on cell biology, biomechanical properties of the membrane, and cell viability should be conducted.

## 1. Introduction

Corneal ulceration is a common ocular disease in veterinary ophthalmology that may lead to numerous complications, including blindness. Surgical treatments are recommended when ulcers have become complicated. Corneal grafting [1] is the most effective procedure for aggressive lesions. Many biological materials, such as the conjunctiva [1], small intestinal submucosa [2], urinary bladder submucosa [3], pericardium [4], amniotic membrane [5,6], and cornea [7], have been reported in use as corneal grafts.

Amniotic membrane (AM) is sourced from various species: humans [6], equine [8], bovine [9], porcine [10], and canine [11]. It is successfully used to treat various human ocular surface disorders [12]. In veterinary practice, clinical use of human AM has been reported in the treatment of cats [13], dogs [14] and horses requiring corneal reconstruction [15].

Ideally, fresh AM would provide the least degradation of membrane properties and structure [16,17]. However, the availability of fresh AM does not align with testing equipment accessibility when there is demand for immediate transplant membrane. Medically accepted preservation methods of AM have been developed as alternatives to allow for a steady supply and pathogen testing. The standard method for human AM, as recommended by the U.S. Food and Drug Administration, is cryopreservation [18,19,20]. Properties of cryopreserved human AM, including thickness, basement membrane components, and its ability to function as epithelial cell cultivation medium, have been documented to be the preeminent available preservation method [18,21]. Sterility, histology, and biological properties of human AM were shown to not be significantly impaired after long term cryopreservation up to 24 months [22]. Currently, there is no standard recommendation for preservation specific to canine AM (CAM).

Histological layers of the CAM are comparable to human AM. However, CAM is not attached to the chorion, and it forms blood vessels at a later stage of pregnancy near the umbilical cord [23]. Cryopreserved CAM has become a potential alternative material for animal treatment. According to previous research, CAM was an effective grafting material to treat keratomalacia in dogs, ankyloblepharon in cats, post-removal of a corneal mass in dogs [11] and cats [24], and deep ulcer in dogs [25]. It can be successfully cultivated with canine corneal epithelial cells, suggesting that CAM is an effective scaffolding material for epithelial cell support [26].

Biomechanical properties such as tensile strength and transparency of a transplant material are important for corneal reconstruction purposes [27], and they are affected by methods of preservation [28,29]. A previous study had shown no difference of tensile strength between fresh human AM, glycerol-cryopreserved human AM, and cryopreserved human AM without storage medium for up to 6 months. However, tensile strength of human AM of the same experiment was shown to increase in the longer preserved groups [17]. The AM of human, porcine, equine, and ovine species have shown to generate J-shaped stress–strain curves, as typically found in soft tissue [30,31] due to the collagen- and elastin-rich stroma. Tensile properties of AM vary by species. Among these, species with AM of the highest loading capacities are equine and human, while those with the highest elasticity of AM are porcine and ovine [30]. Transparency of human AM was investigated and compared between cryopreserved and freeze-dried membranes. Freeze-drying human AM resulted in a more transparent material than cryopreservation [32].

The viability of epithelial cells in human AM is significantly lost in most preserved tissue [16,33]. Glycerol was shown to degenerate cells in preserved human AM. According to the same experiment, time of storage was also shown to be a significant factor of cell viability reduction up to 6 months [17]. Since cell viability may increase immunogenicity and induce inflammation, lower cell viability is desired in transplant material [18]. Trypan blue stain has been demonstrated to be a good method to test cell viability of AM [16,33].

While preserved AM structures of several species have been characterized, there have been no detailed studies of CAM—until now. This study is aimed to investigate and characterize three properties of cryopreserved CAM, namely tensile strength, transparency, and cell viability influenced by storage time for 7 and 30 days.

## 2. Materials and Methods

### 2.1. Canine Amniotic Membranes

Canine amniotic membranes from healthy puppies (n = 36) were collected from pregnant females with completed vaccination programs. They underwent Caesarean sections at the Small Animal Teaching Hospital, Faculty of Veterinary Science, Chulalongkorn University. Females with systemic inflammation within 3 months, history of abortion or dead fetus, high white blood cell count, or signs of inflammation of CAM were excluded.

### 2.2. Media

Washing and storage solutions were freshly prepared in a laminar flow hood with pre-autoclaved equipment. The washing solution was mixed with pre-autoclaved 0.01 M phosphate buffer solution (Gibthai, Bangkok, Thailand), 1 mL of 5-5-10 mg/mL Penicillin-Streptomycin-Neomycin antibiotic mixture (PSN) (Gibthai, Thailand), and 0.5 mL of 5 mg/mL Amphotericin B (Gibthai, Thailand). The storage solution was prepared by mixing 50 mL of pre-autoclaved glycerol (ChemEx, Bangkok, Thailand), 50 mL of DMEM (Gibthai, Thailand), 1 mL of PSN, and 0.02 mL of Amphotericin B. The storage solution was stored at 4 °C until use.

### 2.3. Sample Collection

After a sterile area was prepared, the washing solution and a piece of gauze were laid on a collection tray at the surgical site. Instantly after a removal of a puppy from the uterus, the puppy was separated from the fetal membrane. The membrane was placed on the prepared tray and immediately transported to a laminar flow hood in an insulated box with ice packs. All procedures were completed using aseptic technique.

### 2.4. Cryopreservation

In the laminar flow hood, CAM was separated from the fetal membrane and cleansed with the washing solution. Autoclaved nitrocellulose paper was cut to different sizes: 5 × 2 cm^2^ for tensile strength test, 4 × 4 cm^2^ for transparency test, and 2 × 2 cm^2^ for cell viability test. The cut pieces of nitrocellulose paper were attached to the non-vascularized part of CAM by the stromal side, avoiding the vascularized umbilical cord area. The samples were anatomically located similarly on all CAMs, with the tensile sample located the furthest from the umbilical cord. The CAM samples attached to nitrocellulose membrane were cut and submerged separately in storage solution in a collection vial. All samples were randomly grouped to be cryopreserved at −80 °C for either 7 (7 d CAM; n = 18) or 30 days (30 d CAM; n = 18). After storage, samples were thawed at room temperature for 30 min and cleansed with washing solution before tested.

### 2.5. Tensile Strength Test

The 5 × 2 cm^2^ CAM sample was mounted to a universal testing machine (UTM, Shimadzu, Kyoto, Japan). The nitrocellulose paper was cut across. Tensile strength test was performed automatically by a computerized program (Trapezium 2, Shimadzu, Japan), which was set to pull the membrane apart vertically at the rate of 5 mm/min. The pulling force was measured and recorded every 0.05 s until the CAM fractured.

After a biological stress–strain curve was generated, values of tensile strength included maximum stress (megapascal, MPa), extensibility, Young’s modulus (MPa), length of toe region, and length of linear region. Young’s modulus constant was calculated from the slope of the stress–strain curve at the linear part by Equation (1).

Young’s modulus = Stress/Strain(1)

### 2.6. Transparency Test

The 4 × 4 cm^2^ CAM sample was carefully stripped away from the nitrocellulose paper. It was then loaded onto the transmission compartment of a spectrophotometer (Ultrascan Pro, HunterLab, Reston, VA, USA) by using a kraft paper, a clip, and a plastic stand to hold its shape. Quantitative measurement of direct and diffuse transmission of light was automatically performed using total transmittance mode of the software (EasyMatch QC ver. 4.88.03, HunterLab, Reston, VA, USA).

The transparency test results were reported as a percentage of total transmitted light to incident light (%TTRAN) and percentage of diffusely transmitted light to total transmitted light (%Haze); %TTRAN and %Haze were automatically calculated by Equations (2) and (3), respectively.

%TTRAN = [(I_l_+ I_d_)/I_o_] × 100 (2)

%Haze = [I_d_/(I_l_ + I_d_)] × 100 (3)

### 2.7. Cell Viability Test

After the 2 × 2 cm^2^ CAM sample was stripped away from the nitrocellulose paper, it was mounted on a microscopic slide with the epithelium side up, stained with 20 μL of trypan blue and incubated for 3 min. A sample was visualized under a light microscope. Stained cells represented non-viable cells, while unstained cells represented viable cells. From each CAM sample, microphotographs were randomly taken at 5 fields. The percentage of viable cells compared to all cells (%Viability) was reported as in Equation (4).

%Viability = (Unstained cell count/Total cell count) × 100(4)

### 2.8. Data Analysis

All tested parameters, including those of the tensile strength test (maximum stress, extensibility, Young’s modulus, length of toe region, and length of linear region), transparency test (%TTRAN and %Haze), and cell viability test (%Viability) between 7 d CAM and 30 d CAM, were statistically compared by Mann–Whitney U test (SPSS version 22; IBM, Armonk, NY, USA) with a significance level of *p* < 0.05. Since we did not make any assumptions about the parameters, a non-parametric test was selected for this study.

## 3. Results

### 3.1. Tensile Strength Test

**Figure 1:**
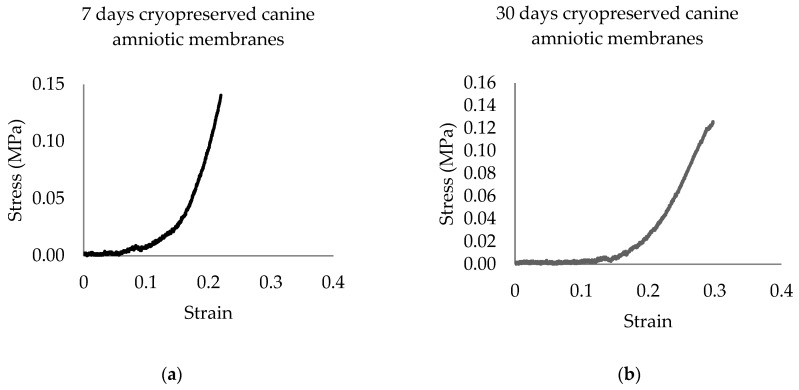
Representative stress–strain curves (MPa) of canine amniotic membranes after: (**a**) 7 days; (**b**) 30 days of cryopreservation.

**Figure 2:**
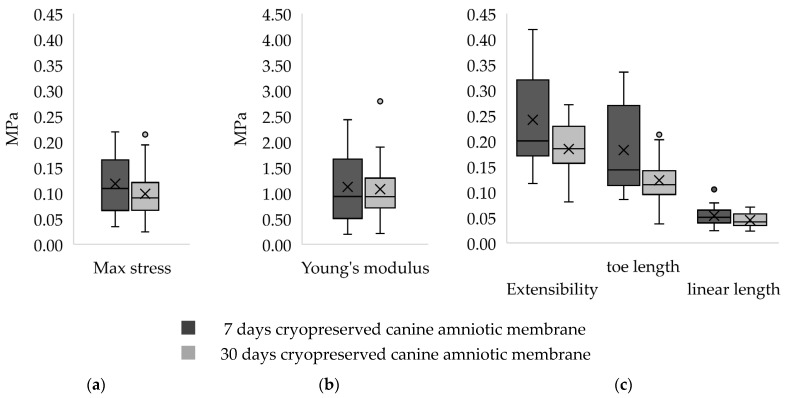
Series of box and whisker plots representing distribution of: (**a**) maximum stress (MPa); (**b**) Young’s modulus (MPa); (**c**) extensibility, toe length, and linear length of canine amniotic membranes after 7 days and 30 days of cryopreservation. The plots show skewed distribution as the mean values (X marks) are not equal to the median values (middle lines) with a few outliers (o marks). The boxes represent the interquartile range while the ends of whiskers represent the maximum and minimum value of the data.

The stress–strain curves of all CAM samples matched the J-shaped stress–strain curve of biological material. Representative stress–strain curves of the two groups are shown in Figure 1. A higher range of data distribution was observed in a group of 7 d CAM samples with less symmetry, as compared to the other as shown in Figure 2. Right-skewed data distributions were noted. The median of maximum stress capacity, extensibility, and Young’s modulus were higher in 7 d CAM samples as compared to 30 d sample (Table 1), though they were not statistically significant.

### 3.2. Transparency Test

**Figure 3:**
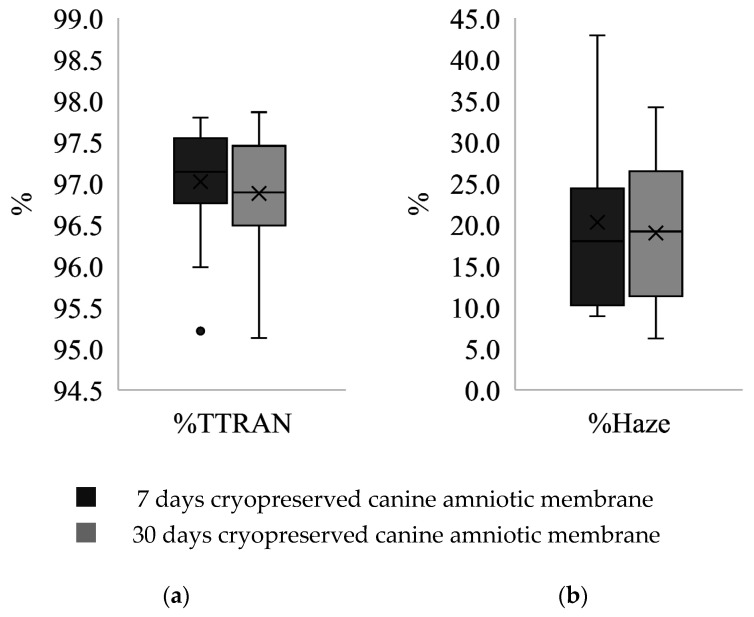
Series of box and whisker plots representing distribution of: (**a**) %TTRAN; (**b**) %Haze of canine amniotic membranes (CAM) after cryopreservation for 7 days and 30 days. The plots of CAM after 7 days of cryopreservation show skewed distribution as the mean values (X marks) are not equal to the median values (middle lines) with a few outliers (o marks), while the plots of CAM after 30 days of cryopreservation are more normally distributed. The boxes represent the interquartile range while the ends of whiskers represent the maximum and minimum values of the data.

The range of percentage of total transmitted light to incident light (%TTRAN) and diffusely transmitted light to total transmitted light (%Haze) of the 30 d CAM samples was more dispersed compared to the 7 d CAM samples, though more data symmetry was observed (Figure 3). The median %TTRAN was 97.14 ± 0.78 and 96.89 ± 0.96 in a group of 7 d and 30 d CAM samples, respectively. In contrast, the median %Haze was 17.99 ± 14.17 in a 7 d cryopreserved group, and 19.16 ± 15.08 in the other. The differences were not statistically significant.

### 3.3. Cell Viability Test

**Figure 4:**
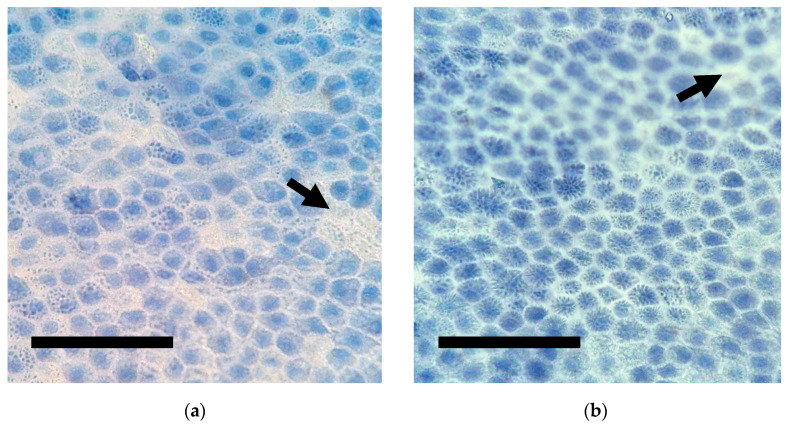
Representative microphotographs of trypan blue-stained single layer of epithelium of canine amniotic membranes after: (**a**) 7 days; (**b**) 30 days of cryopreservation. Note the unstained viable cells (black arrows). (Scale bar = 100 micron).

Sheets of uniformly arranged polygonal epithelial cells of CAM were observed (Figure 4). Epithelial cells were tightly packed with distinct cell margins. Some cells showed homogeneous staining of cytoplasm, while the accumulation of multiple vacuoles with trypan blue was observed in some other cells. Round eccentric nuclei were more intensely stained than the cytoplasm. Unstained cells were sparsely observed. The median percentages of viable cells of the 7 d CAM and 30 d CAM were 8.77 ± 11.39 and 1.75 ± 4.70, respectively. Cell viability of CAM cryopreserved for 7 days was statistically higher than that of 30 days (*p* = 0.037).

## 4. Discussion

This is the first report of the biomechanical properties of CAM. Stress–strain curves generated by CAM matched the J-shaped curve demonstrated in AM of other species, including human [27], porcine [34], ovine, and equine [30]. This type of stress–strain curve is typically found in biological tissues containing collagen and elastin that build up a three-dimensional network such as skin, tendon, and blood vessels [31]. All parameters of tensile strength were non-statistically lower in CAM cryopreserved for 30 days. This suggests that longer duration of cryopreservation may mildly damage the cross-linking bond of elastin, resulting in a deterioration of its tensile strength properties [35]. Transparency properties of cryopreserved CAM were well maintained in both groups (Figure 3), suggesting cryopreservation is a good method to preserve CAM for optical transplants. Our finding is consistent with a prior study, which stated that transparency is preserved in human AM that had undergone repeated freezing procedures of cryopreservation up to two times [36].

The biomechanical properties of cryopreserved AM from humans and pigs have been reported [34,37]. When comparing the AM of humans, pigs, and dogs, CAM exhibits the lowest maximum stress capacity, which indicates the least endurance against applied force. Among various species studied, CAM exhibits the least Young’s modulus, indicating the lowest stiffness. It also displays the low extensibility referring to the least endurance against deformation. Differences between the anatomical part of the membrane [38] and sample geometries [39] should be considered for different membrane thickness. Clinical application of CAM for corneal reconstruction in veterinary practice suggests using the multilayer suturing technique to increase tensile strength for tectonic purposes. In one study, human AM was applied in patients with various depths of corneal damage. In the group of corneal perforation, the bottom of the perforated site was covered with a sheet of human AM. Layers of membrane were filled up in the thinning area, followed by the large piece of membrane covering the entire corneal defect. It revealed that not only can multiple human AM restore corneal stromal thickness, but that the membranes became gradually transparent, resulting in a clearer healing area similar to the adjacent area [40]. The cross-linking technique by lamination of eight layered human AM offers better transparency than multiple layer AM transplant shown by quantitative evaluation of light transmittance [28]. Regarding the transparency, the crossing-linking technique increased light transmittance and enhanced the tensile strength of human AM [37]. Therefore, cross-linking techniques on CAM should be further studied to explore the possibility of creating a similar transparency and resilience for CAM. Further research on the transparency of multiple layered CAM is suggested to demonstrate whether the membrane transparency is retained. Effects of multiple freeze-thaw cycles to CAM transparency may provide information mimicking clinical use, especially for tissue reconstruction.

We found cell viability is poorly preserved in cryopreserved CAM, which is consistent with many previous studies with different measurement methods [16,17,33,41,42]. A comparison study of cell viability of cryopreserved human AM using a luminescence cell viability assay by Wagner and others (2018) revealed an intense decrease of metabolic cell activity of preserved AM in −80 °C. Luminescence signals of tissue preserved in glycerol at 14 days and 30 days were 67.90 and 49.12, respectively, which were significantly reduced compared to 238.56 of fresh AM [17]. Another study by Hennerbichler and others (2007) measured cell viability by EZ4U cell proliferation and cytotoxicity assay, as well as trypan blue staining. Both methods showed consistent results of intense cell viability reduction similar to our findings. They demonstrated that by 21 days of cryopreservation, cell viability was diminished to 13–18%, which was more pronounced than AM that was stored above 0 °C for 28 days (15–35%) [33]. Similarly, evaluations of cell viability of fresh and cryopreserved human AM by trypan blue and EZ4U cell proliferation and cytotoxicity assay [16] and MTT assay imaged with confocal microscopy [41] showed that cryopreserved AM had marked reduction of cell viability. Moreover, no live cells were identified in any AM commercial products preserved by freeze-dried method, dehydration, or cryopreservation [42]. Dramatic reduction of cell viability demonstrated in our result as well as studies of human AM, implies that cryopreserved AM are not suitable for regenerative medicine purposes. Nonetheless, cryopreservation of the membrane reduced risk of infectious contamination. Meanwhile, various growth factors, collagen proteins and basement membrane were not significantly impaired up to 24 months [22]. These characteristics of cryopreserved AM are clinically beneficial as a scaffold biomaterial.

Several factors play important roles for clinical selection of membranes for transplantation. Sufficient tissue strength of CAM preserved up to 30 days offers comparable support to corneal damage as the samples preserved for 7 days. Non-significant transparency between the two groups confirms good optical purpose when transplanted. Therefore, we have shown that cryopreservation of CAM between 7 and 30 days resulted in similar properties for both optical and tectonic purposes. Our first investigation of the basic biomechanical properties and cell viability of cryopreserved CAM provides the data to allow future studies to develop this promising material not only for veterinary practice, but also tissue banking. Further study is needed to increase cell viability by focusing on time of tissue storage, storage methods, and media for regenerative therapy. Investigation of longer CAM cryopreservation time is suggested as cryopreserved human AM has been proven to be effective transplant material after being stored for up to 6 months [43]. Furthermore, it will be interesting to explore the presence of growth factors and cytokines to determine the therapeutic benefit of CAM. The anatomical location of CAM and breed of dogs may be factors causing wide variations in this study’s results. There are reports of thickness variation among individuals and anatomical locations in humans [44]. While there is no data on CAM, the heterogeneous nature of AM as demonstrated in humans was taken into consideration during this study’s sample collection by selecting similar areas, and the umbilical cord area was generally avoided due to the vasculature. There were limitations to this study that included the emergency nature of cesarean surgery, the unpredictable breed of donor dogs, and limited equipment availability which led to inaccessible fresh CAM information. There may be opportunities in the future to obtain baseline data from fresh CAM.

Considering that CAM is an easily available material in a veterinary hospital setting, having information about its basic properties is crucial for creating a more readily available transplantation material to meet its demand at a lower cost, thus improving animal care in veterinary medicine.

## 5. Conclusions

Tensile strength and transparency are essential properties of CAM, which are required when determining viable corneal transplant materials. These properties of CAM preserved from 7 to 30 days can provide mechanical stability and transparency for the application in veterinary ophthalmology.

While the cell viability of CAM is not well preserved by cryopreservation, it is a good processing method for creating a CAM for scaffolding purpose with low graft rejection risk. As for a regenerative medicine purpose, more study is needed to increase cell survival.

## Figures and Tables

**Table 1:** Median ± interquartile range of tensile strength parameters of canine amniotic membranes that were cryopreserved for 7 days and 30 days.

Parameter/Canine Amniotic Membrane	7 Days ^1^	30 Days ^1^
Maximum stress (MPa)	0.11 ± 0.10	0.09 ± 0.05
Extensibility	0.29 ± 0.15	0.19 ± 0.07
Young’s Modulus (MPa)	0.93 ± 1.16	0.11 ± 0.05
Toe region length	0.14 ± 0.16	0.11 ± 0.05
Linear region length	0.05 ± 0.03	0.04 ± 0.02

^1^ days in cryopreservation.

## Data Availability

Not applicable.
